# Redetermination of 2-methyl-4-nitro­pyridine *N*-oxide

**DOI:** 10.1107/S1600536814004450

**Published:** 2014-03-12

**Authors:** Max Peukert, Wilhelm Seichter, Edwin Weber

**Affiliations:** a Institut für Organische Chemie, TU Bergakademie Freiberg, Leipziger Strasse 29, D-09596 Freiberg/Sachsen, Germany

## Abstract

An improved crystal structure of the title compound, C_6_H_6_N_2_O_3_, is reported. The structure, previously solved [Li *et al.* (1987 ▶). *Jiegou Huaxue (Chin. J. Struct. Chem.)*, **6**, 20–24] in the ortho­rhom­bic space group *Pca*2_1_ and refined to *R* = 0.067, has been solved in the ortho­rhom­bic space group *Pbcm* with data of enhanced quality, giving an improved structure (*R* = 0.0485). The mol­ecule adopts a planar conformation with all atoms lying on a mirror plane. The crystal structure is composed of mol­ecular sheets extending parallel to the *ab* plane and connected *via* C—H⋯O contacts involving ring H atoms and O atoms of the *N*-oxide and nitro groups, while van der Waals forces consolidate the stacking of the layers.

## Related literature   

For the synthesis and preparative aspects of pyridine-*N*-oxides, see: Fontenas *et al.* (1995 ▶); Katritzky & Lagowski (1971 ▶); Kilenyi (2001 ▶); Mosher *et al.* (1963 ▶). For the preparation of the title compound, see: Ashimori *et al.* (1990 ▶) and for potential applications, see: Elemans *et al.* (2009 ▶); Weber & Vögtle (1976 ▶); Winter *et al.* (2004 ▶). For the previous report of its crystal structure, see: Li *et al.* (1987 ▶). For non-classical hydrogen bonds, see: Desiraju & Steiner (1999 ▶).
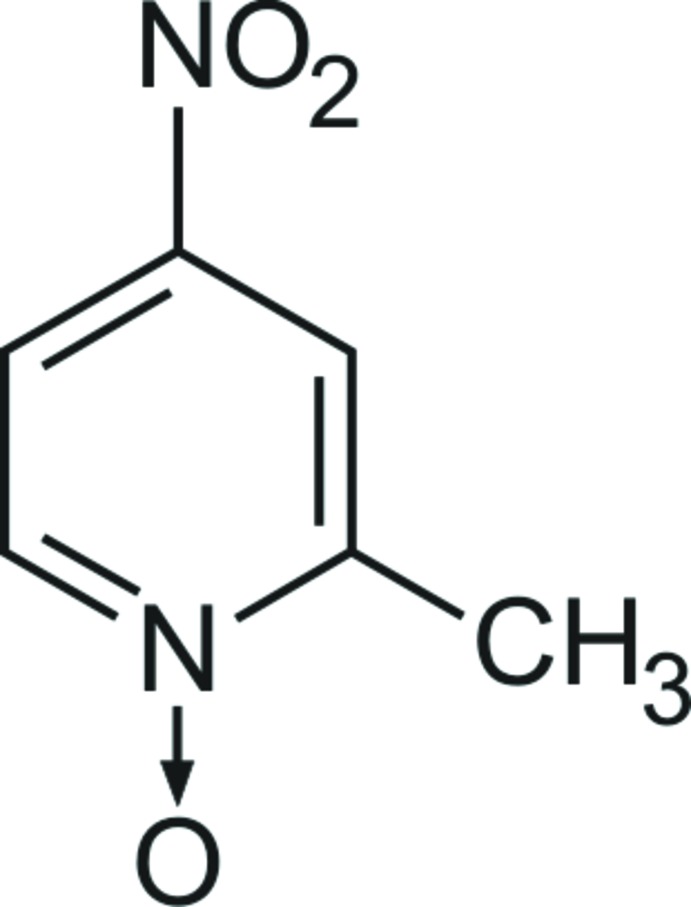


## Experimental   

### 

#### Crystal data  

C_6_H_6_N_2_O_3_
*M* *_r_* = 154.13
Orthorhombic, 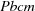

*a* = 8.6775 (7) Å
*b* = 12.4069 (10) Å
*c* = 6.1995 (5) Å
*V* = 667.44 (9) Å^3^
*Z* = 4
Mo *K*α radiation
μ = 0.13 mm^−1^
*T* = 153 K
0.57 × 0.30 × 0.23 mm

#### Data collection  

Bruker APEXII CCD area-detector diffractometer
Absorption correction: multi-scan (*SADABS*; Bruker, 2008 ▶) *T*_min_ = 0.932, *T*_max_ = 0.972
19832 measured reflections
1100 independent reflections
973 reflections with *I* > 2σ(*I*)
*R*_int_ = 0.028

#### Refinement  

*R*[*F* ^2^ > 2σ(*F* ^2^)] = 0.049
*wR*(*F* ^2^) = 0.147
*S* = 1.10
1100 reflections
74 parameters
H atoms treated by a mixture of independent and constrained refinement
Δρ_max_ = 0.36 e Å^−3^
Δρ_min_ = −0.34 e Å^−3^

### 

Data collection: *APEX2* (Bruker, 2008 ▶); cell refinement: *SAINT-NT* (Bruker, 2008 ▶); data reduction: *SAINT-NT*; program(s) used to solve structure: *SHELXS97* (Sheldrick, 2008 ▶); program(s) used to refine structure: *SHELXL97* (Sheldrick, 2008 ▶); molecular graphics: *ORTEP-3 for Windows* (Farrugia, 2012 ▶); software used to prepare material for publication: *SHELXL97*.

## Supplementary Material

Crystal structure: contains datablock(s) I, New_Global_Publ_Block. DOI: 10.1107/S1600536814004450/zp2011sup1.cif

Structure factors: contains datablock(s) I. DOI: 10.1107/S1600536814004450/zp2011Isup2.hkl

Supporting information file. DOI: 10.1107/S1600536814004450/zp2011Isup3.cml

CCDC reference: 988898

Additional supporting information:  crystallographic information; 3D view; checkCIF report

## Figures and Tables

**Table 1:** Hydrogen-bond geometry (Å, °)

*D*—H⋯*A*	*D*—H	H⋯*A*	*D*⋯*A*	*D*—H⋯*A*
C2—H2*A*⋯O1^i^	0.95	2.29	3.225 (2)	169
C5—H5⋯O2^ii^	0.95	2.36	3.301 (2)	173

Symmetry codes: (i) 
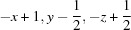
; (ii) 
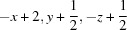
.

**Fig. 1.**
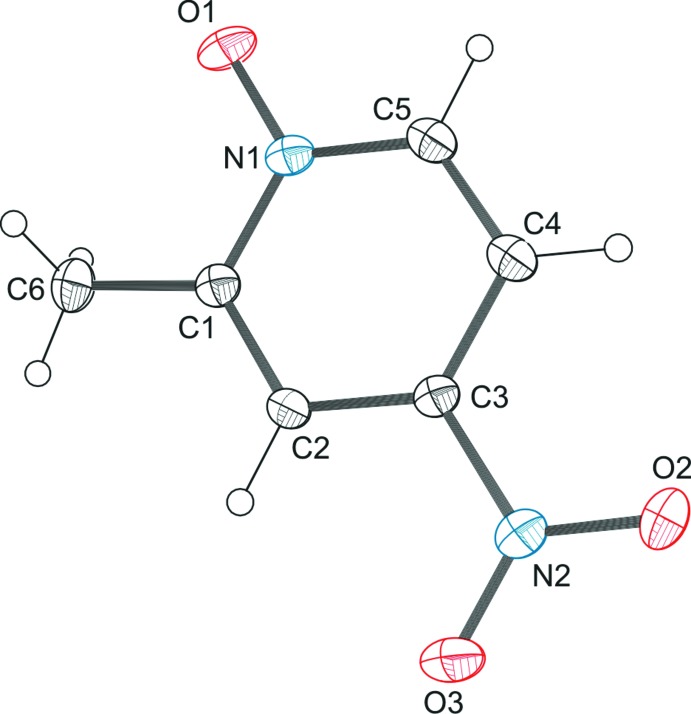
Perspective view of the molecular structure of the title compound including the atom numbering. Anisotropic displacement parameters for non-hydrogen atoms are drawn at a 50% probability level.

**Fig. 2.**
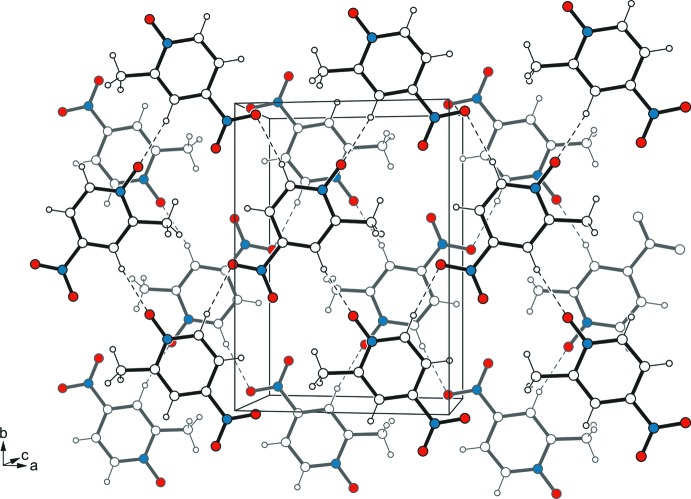
Packing diagram of the title compound viewed down the c-axis. Hydrogen bonds are displayed as broken lines.

## supplementary crystallographic information

### 1. Comment

Pyridine *N*-oxides are readily formed by oxidation of corresponding pyridines (Kilenyi, 2001; Mosher *et al.*, 1963). In contrast to the simple pyridines they facilitate an electrophilic substitution reaction in the ring position-4, hence being important intermediates in the synthesis of pyridine derivatives featuring a complex substitution pattern (Katritzky & Lagowski, 1971). Moreover, when 2-methylpyridine *N*-oxides are treated with trifluoroacetic anhydride, the Boekelheide reaction occurs to give 2-(hydroxymethyl)pyridines (Fontenas *et al.*, 1995) which are of relevance to make available chelating (Winter *et al.*, 2004), macrocyclic (Weber & Vögtle, 1976) and linker-type (Elemans *et al.*, 2009) ligands. In the course of a respective synthesis of the latter kind, the title compound was prepared and its structure redetermined. The previous crystal structure of the compound (reported in 1987 by Li *et al.*) has been solved in the orthorhombic space group *Pca*2_1_ and refined to an *R*-value of 6.7%. The repeated analysis of the crystal structure with data of enhanced quality yields a crystal structure of space group *Pbcm* with nearly identical cell dimensions. The centrosymmetry of the crystal structure is sustained by the statistical analysis of E-values. The molecule is located on the crystallographic symmetry plane and thus adopts perfect planarity (Fig. 1). According to this, two-dimensional supramolecular aggregates extending parallel to the crystallographic *ab*-plane and with the molecules connected *via* C—H···O hydrogen bonding (Desiraju & Steiner, 1999) that involves ring H atoms and both O atoms of the *N*-oxide (C—H···O_N-oxide_ 2.29 Å, 169 °) and nitro groups (*C*—*H*···O_nitro_ 2.36 Å, 173 °) represent the basic entities of the crystal structure (Fig. 2). As no other type of intermolecular interactions are observed, the crystal structure is stabilized by van der Waals forces in direction of the stacking axes of the molecular sheets.

### 2. Experimental

The title compound was synthesized *via* nitration of 2-methylpyridine *N*-oxide following a described procedure (Ashimori *et al.*, 1990). Crystallization from toluene/chloroform (1/1) yielded yellow needles which were used for X-ray single-crystal structure analysis.

### 3. Refinement

Aromatic H atoms were positioned geometrically and allowed to ride on their parent atoms, with C—H = 0.95 Å and *U*_iso_ = 1.2 *U*_eq_(C).

### Crystal data

**Table:**

C_6_H_6_N_2_O_3_	*F*(000) = 320
*M**_r_* = 154.13	*D*_x_ = 1.534 Mg m^−^^3^
Orthorhombic, *P**b**c**m*	Mo *K*α radiation, λ = 0.71073 Å
Hall symbol: -P 2c 2b	Cell parameters from 6590 reflections
*a* = 8.6775 (7) Å	θ = 2.4–35.0°
*b* = 12.4069 (10) Å	µ = 0.13 mm^−^^1^
*c* = 6.1995 (5) Å	*T* = 153 K
*V* = 667.44 (9) Å^3^	Column, yellow
*Z* = 4	0.57 × 0.30 × 0.23 mm

### Data collection

**Table:**

Bruker APEXII CCD area-detector diffractometer	1100 independent reflections
Radiation source: fine-focus sealed tube	973 reflections with *I* > 2σ(*I*)
Graphite monochromator	*R*_int_ = 0.028
phi and ω scans	θ_max_ = 30.4°, θ_min_ = 2.9°
Absorption correction: multi-scan (*SADABS*; Bruker, 2008)	*h* = −12→12
*T*_min_ = 0.932, *T*_max_ = 0.972	*k* = −17→17
19832 measured reflections	*l* = −8→8

### Refinement

**Table:**

Refinement on *F*^2^	Secondary atom site location: difference Fourier map
Least-squares matrix: full	Hydrogen site location: inferred from neighbouring sites
*R*[*F*^2^ > 2σ(*F*^2^)] = 0.049	H atoms treated by a mixture of independent and constrained refinement
*wR*(*F*^2^) = 0.147	*w* = 1/[σ^2^(*F*_o_^2^) + (0.0871*P*)^2^ + 0.2298*P*] where *P* = (*F*_o_^2^ + 2*F*_c_^2^)/3
*S* = 1.10	(Δ/σ)_max_ < 0.001
1100 reflections	Δρ_max_ = 0.36 e Å^−^^3^
74 parameters	Δρ_min_ = −0.34 e Å^−^^3^
Primary atom site location: structure-invariant direct methods	

### Special details

**Table:**

Geometry. All e.s.d.'s (except the e.s.d. in the dihedral angle between two l.s. planes) are estimated using the full covariance matrix. The cell e.s.d.'s are taken into account individually in the estimation of e.s.d.'s in distances, angles and torsion angles; correlations between e.s.d.'s in cell parameters are only used when they are defined by crystal symmetry. An approximate (isotropic) treatment of cell e.s.d.'s is used for estimating e.s.d.'s involving l.s. planes.
Refinement. Refinement of *F*^2^ against ALL reflections. The weighted *R*-factor *wR* and goodness of fit *S* are based on *F*^2^, conventional *R*-factors *R* are based on *F*, with *F* set to zero for negative *F*^2^. The threshold expression of *F*^2^ > σ(*F*^2^) is used only for calculating *R*-factors(gt) *etc*. and is not relevant to the choice of reflections for refinement. *R*-factors based on *F*^2^ are statistically about twice as large as those based on *F*, and *R*- factors based on ALL data will be even larger. The C—H bonds of the methyl group were restrained to a target value of 0.89 (1) Å.

### Fractional atomic coordinates and isotropic or equivalent isotropic displacement parameters (Å^2^)

**Table:**

	*x*	*y*	*z*	*U*_iso_*/*U*_eq_	
O1	0.52895 (16)	0.30510 (10)	0.2500	0.0309 (3)	
O2	1.05609 (15)	−0.02519 (12)	0.2500	0.0355 (4)	
O3	0.85937 (17)	−0.13544 (11)	0.2500	0.0441 (4)	
N1	0.61843 (17)	0.22205 (10)	0.2500	0.0223 (3)	
N2	0.91523 (17)	−0.04406 (12)	0.2500	0.0276 (3)	
C1	0.55535 (18)	0.12002 (13)	0.2500	0.0216 (3)	
C2	0.65356 (17)	0.03153 (12)	0.2500	0.0203 (3)	
H2A	0.6128	−0.0395	0.2500	0.024*	
C3	0.81182 (18)	0.04811 (12)	0.2500	0.0214 (3)	
C4	0.87504 (19)	0.15129 (13)	0.2500	0.0249 (3)	
H4	0.9834	0.1621	0.2500	0.030*	
C5	0.77482 (19)	0.23649 (13)	0.2500	0.0247 (3)	
H5	0.8150	0.3077	0.2500	0.030*	
C6	0.38480 (19)	0.11425 (16)	0.2500	0.0283 (4)	
H6A	0.3468 (19)	0.1469 (13)	0.133 (2)	0.040 (5)*	
H6B	0.358 (3)	0.0444 (9)	0.2500	0.035 (6)*	

### Atomic displacement parameters (Å^2^)

**Table:**

	*U*^11^	*U*^22^	*U*^33^	*U*^12^	*U*^13^	*U*^23^
O1	0.0389 (7)	0.0217 (6)	0.0322 (7)	0.0136 (5)	0.000	0.000
O2	0.0209 (6)	0.0365 (7)	0.0492 (8)	0.0072 (5)	0.000	0.000
O3	0.0376 (8)	0.0197 (6)	0.0750 (12)	0.0050 (5)	0.000	0.000
N1	0.0277 (7)	0.0182 (6)	0.0210 (6)	0.0033 (5)	0.000	0.000
N2	0.0254 (6)	0.0243 (7)	0.0329 (7)	0.0049 (5)	0.000	0.000
C1	0.0232 (6)	0.0210 (7)	0.0206 (7)	0.0009 (5)	0.000	0.000
C2	0.0211 (7)	0.0170 (6)	0.0228 (7)	−0.0018 (5)	0.000	0.000
C3	0.0214 (7)	0.0182 (6)	0.0247 (7)	0.0023 (5)	0.000	0.000
C4	0.0272 (7)	0.0215 (7)	0.0259 (7)	−0.0047 (6)	0.000	0.000
C5	0.0286 (7)	0.0211 (7)	0.0244 (7)	−0.0050 (6)	0.000	0.000
C6	0.0202 (7)	0.0350 (9)	0.0296 (8)	0.0013 (6)	0.000	0.000

### Geometric parameters (Å, º)

**Table:**

O1—N1	1.2902 (17)	C2—C3	1.389 (2)
O2—N2	1.244 (2)	C2—H2A	0.9500
O3—N2	1.233 (2)	C3—C4	1.393 (2)
N1—C5	1.369 (2)	C4—C5	1.369 (2)
N1—C1	1.379 (2)	C4—H4	0.9500
N2—C3	1.454 (2)	C5—H5	0.9500
C1—C2	1.390 (2)	C6—H6A	0.892 (9)
C1—C6	1.482 (2)	C6—H6B	0.898 (10)
			
O1—N1—C5	119.48 (14)	C2—C3—C4	121.72 (14)
O1—N1—C1	119.61 (14)	C2—C3—N2	119.61 (14)
C5—N1—C1	120.91 (13)	C4—C3—N2	118.68 (14)
O3—N2—O2	123.99 (15)	C5—C4—C3	117.36 (15)
O3—N2—C3	118.73 (14)	C5—C4—H4	121.3
O2—N2—C3	117.28 (14)	C3—C4—H4	121.3
N1—C1—C2	118.79 (14)	N1—C5—C4	121.92 (14)
N1—C1—C6	116.16 (14)	N1—C5—H5	119.0
C2—C1—C6	125.05 (15)	C4—C5—H5	119.0
C3—C2—C1	119.30 (14)	C1—C6—H6A	110.3 (11)
C3—C2—H2A	120.3	C1—C6—H6B	108.0 (16)
C1—C2—H2A	120.3	H6A—C6—H6B	109.9 (14)
			
O1—N1—C1—C2	180.0	O2—N2—C3—C2	180.0
C5—N1—C1—C2	0.0	O3—N2—C3—C4	180.0
O1—N1—C1—C6	0.0	O2—N2—C3—C4	0.0
C5—N1—C1—C6	180.0	C2—C3—C4—C5	0.0
N1—C1—C2—C3	0.0	N2—C3—C4—C5	180.0
C6—C1—C2—C3	180.0	O1—N1—C5—C4	180.0
C1—C2—C3—C4	0.0	C1—N1—C5—C4	0.0
C1—C2—C3—N2	180.0	C3—C4—C5—N1	0.0
O3—N2—C3—C2	0.0		

### Hydrogen-bond geometry (Å, º)

**Table:**

*D*—H···*A*	*D*—H	H···*A*	*D*···*A*	*D*—H···*A*
C2—H2*A*···O1^i^	0.95	2.29	3.225 (2)	169
C5—H5···O2^ii^	0.95	2.36	3.301 (2)	173

Symmetry codes: (i) −*x*+1, *y*−1/2, −*z*+1/2; (ii) −*x*+2, *y*+1/2, −*z*+1/2.
